# Knowledge, Attitudes, and Practice Toward Prevention of COVID-19 Among Jimma Town Residents: A Community-Based Cross-Sectional Study

**DOI:** 10.3389/fpubh.2022.822116

**Published:** 2022-04-27

**Authors:** Iyasu Tadesse Bukata, Lelisa Sena Dadi, Andualem Mossie Ayana, Demelash Mengistu, Delnesa Yewal, Tariku Sime Gizaw, Yohannes Markos Woldesenbet

**Affiliations:** ^1^Department of Biomedical Sciences, Faculty of Medical Sciences, Jimma University, Jimma, Ethiopia; ^2^Department of Epidemiology, Faculty of Public Health, Jimma University, Jimma, Ethiopia; ^3^Department of Media and Communication Studies, College of Social Sciences, Jimma University, Jimma, Ethiopia; ^4^Department of Medical Laboratory Sciences, Faculty of Medical Sciences, Jimma University, Jimma, Ethiopia; ^5^School of Medicine, Wolayita Sodo University, WolaitaSodo, Ethiopia

**Keywords:** knowledge, attitude, practice, COVID-19, Jimma, Ethiopia

## Abstract

**Background:**

Coronavirus disease (COVID-19) was first reported by the World Health Organization on 31 December 2019, and later, it was declared a global pandemic on 12 March 2020. To date, it is a great challenge to the world including Ethiopia. Therefore, to attain effective prevention and control of the COVID-19 pandemic, improving the knowledge, attitude, and practices of the community is necessary.

**Objective:**

To assess, knowledge, attitudes, and practice, and associated factors of COVID-19 among Jimma Town residents.

**Methods:**

A community-based cross-sectional study was conducted among 1,500 Jimma town residents from May through June 2020. Adults aged ≥18 years were included in the study. Data were collected using a structured questionnaire that was adopted from different literature. A face-to-face interview was implemented to collect data. Analysis was done by using SPSS version 22. *p* < 0.05 was used to declare statistical significance.

**Result:**

A total of 1,500 participants were enrolled in the study. The majority of the respondents were female (59.3%). About 841 (56.1%) of the participants had knowledge about COVID-19. Educational status, household wealth index, and employment showed association with knowledge of COVID-19. Government-owned television (37.3%) was the primary source of information about COVID-19 in the Jimma population. Only 46.6% of respondents had good attitudes toward the COVID-19 pandemic and about 638 (42.5%) of the study participants had good practice toward COVID-19. The mean practice score was 1.98 (± 0.319). Study participants who were residing in the outskirts of the town were 0.37 less likely to apply good practice regarding COVID-19 prevention measures than those around the center of the town. Whereas, households with a family size of four to five individuals were 1.4 times more likely to show good practice against COVID-19 compared to households with ≤3 individuals (AOR: 1.41; CI: 1.05, 1.91).

**Conclusion:**

Jimma town community has low knowledge, attitude, and practice regarding COVID-19. Knowledge, attitude, and practice scores regarding COVID-19 are significantly related to educational status, being self-employed, occupation, marital status, residence, family size, and household relative wealth index. Preventive health advisories to upraise knowledge, attitude, and practice are crucial to prevent and control COVID-19.

## Introduction

Coronavirus disease (COVID-19) is a global pandemic that is caused by severe acute respiratory syndrome coronavirus-2 (SARS-CoV-2). It was first seen in Wuhan, Hubei Province, China, on 31 December 2019, and later, on 12 March 2020, it was declared by World Health Organization (WHO) as a global pandemic (1–4). The disease is highly infectious, and its main clinical symptoms include fever, dry cough, fatigue, myalgia, and dyspnea. In China, 18.5% of the patients with COVID-19 develop to the severe stage, which is characterized by acute respiratory distress syndrome, septic shock, difficult-to-tackle metabolic acidosis, and bleeding and coagulation dysfunction (5, 6).

According to the WHO report on COVID 19, as of 28 November 2021, there were 260, 493,573 confirmed cases, and 5, 195, 354 confirmed deaths globally^1^. Data also indicate that infection and death from the pandemic are increasing highly despite the battle against it is still continuing (7). Yet, the use of face masks, good hand hygiene, and social distancing as community interventions are effective to control the spread of the pandemic (8). In order to familiarize effective control measures, having knowledge about basic hygiene principles and modes of disease transmission, and measures in such an environment is, vitally important (3). Therefore, improving the knowledge, attitude, and practices of the community is essential (9). The effectiveness of community interventions in lower and middle income countries should be informed by adherence to the mitigation measures and contextual factors taking into account the best practices (8).

The level of knowledge, attitude, and practices of the community regarding COVID 19 vary with the geographical site in the world. In the rural part of Ethiopia, the communities' residents may notice themselves as low risk of acquiring COVID-19, and negligence of the prevention method is visible community gathering is experienced as usual. Therefore, assessing knowledge, attitude, and practice of COVID-19 prevention and implementing interventions is mandatory in the different geographic areas of the country. To know the level of knowledge, there is no study conducted in the Jimma community to assess the level of knowledge, attitude, and practice of the community. Therefore, the current study is aimed to assess this gap and develop an intervention strategy to alleviate the burden of the problem in the studied area.

## Methods and Materials

### Study Design and Area

A community-based cross-sectional study was conducted in Jimma Town, Ethiopia, among adults aged 18 years and beyond to assess their knowledge, attitude, and practice toward the prevention of COVID-19. The study was conducted from the 1st week of May through the last week of June 2020. Jimma is the largest city in southwestern Ethiopia and consists of 17 Kebeles (the lowest administrative unit). The town is located 352Km from Addis Ababa, the capital city of Ethiopia, and has a latitude and longitude of 7°40′N 36°50′E. According to the Oromia region population projection, the town has nearly 207,000 residents. There are two public and two private hospitals, and five health centers in the town.

### Study Population and Target Sample Size

Sample size was determined using single population proportion formula (n = [Zα/2] 2 p [1-p]/d^2^). We used a *p*-value of 50% and a 0.5 margin of error. Using this calculation we got a sample size of 384.

But, to reach an interviewee in the households, we needed to go through four strata/ stages: Jimma town, sub-cities, kebeles, households, and individuals. Hence, we implemented a multi-stage sampling technique that is indebted to the use of a design effect. Therefore, after considering a design effect of 4.0 and a non-response rate of 10%, we got a sample size of 1,690. But, after data collection about 200 questionnaires were below quality. Hence, these questionnaires were discarded and 1,500 data were used for analysis.

### Participant Recruitment Procedure

We selected six Kebeles from the 17 kebeles in the town. The selection of the Kebeles was done using simple random sampling. The six kebeles are about 30% of the whole town. A systematic sampling technique was undertaken to identify the households in each Kebele. An individual from the household was then chosen using a lottery method.

### Data Collection Tool

Data collection was done by face-to-face interview using a structured questionnaire which is developed after reviewing several articles that were done to measure KAP toward COVID-19 and published in reputable journals. The questionnaire consisted of 51 close-ended questions that aimed to collect information on socio-demographic characteristics, travel history, and KAP related to COVID-19 from the study respondents.

Data collectors and supervisors were given 2 days of training on the nature of the study and WHO-recommended precaution measures related to COVID-19.

The interview process took ~10–15 min to complete. The data collection tool was initially prepared in English version followed by translation to local Afan Oromo and Amharic languages. Consistency of content, clarity, and appropriate meaning between the three versions were maintained through back-translation of the questionnaire to the original version. Additionally, the practicability, validity, and interpretability of answers for the respective questions were confirmed by performing a pre-test in 10% of the targeted sample size. Based on this pre-test study, the format and wording of questions were corrected and refined.

### Statistical Analysis

Data were checked for completeness. Data entry was done using EPI data version 3.1 and exported to SPSS for Windows version 22 for analysis.

## Results

### Sociodemographic Characteristics

A total of 1,500 participants were enrolled in the study. The majority of the respondents were female (59.3%) and about 34.9% of the respondents were between 30 and 39 years of age. The mean age of study participants was 35.8 (standard deviation of ± 11.6). With regard to the residence of the respondents, most (81.8%) of them were living in Jimma town and married (76.8%). The majority of study participants had only primary school educational achievement, and most of the study participants had a family size of four to six individuals per household and in the 2nd /4th quintiles of household relative wealth index (Table 1).

**Table 1:** Socio-demographic characteristics of study participants, Jimma Town, Southwest Ethiopia, 2020.

**Variables**	**Categories**	**Number**	**Percent**
Sex	Male	611	(40.7)
	Female	889	59.3
	**Total**	**1,500**	**100**
Age in years	<20 years	68	4.5
	20–29 years	407	27.1
	30–39 years	523	34.9
	40–49 years	293	19.5
	50–59 years	133	8.9
	≥60 years	76	5.1
	Mean (± St. Dev.)	35.8 (11.6)	
	**Total**	**1,500**	**100**
Marital status	Married	1,152	76.8
	Single	210	14.0
	Widowed	86	5.7
	Divorced/separated	52	3.5
	**Total**	**1,500**	**100**
Educational status	Illiterate	258	17.2
	Read and write	308	20.5
	Primary school	427	28.5
	Secondary school	278	18.5
	Diploma	134	8.9
	Degree or above	95	6.3
	**Total**	**1,500**	**100**
Occupational status	Housewife	538	35.9
	Farmer	288	19.2
	Private	181	12.1
	Civil servant	165	11.0
	Self-employee	150	10.0
	Student	117	7.8
	Other	61	4.1
	**Total**	**1,500**	**100**
Residence	Jimma Town	1,227	81.8
	Suburbs	273	18.2
	**Total**	**1,500**	**100**
Household size	≤3 individuals	400	27.0
	4–5 individuals	620	41.8
	≥ 6 individuals	462	31.2
	**Total**	**1,482**	**100**
Household relative wealth index	1st quantile (poorest)	282	18.8
	2nd quantile (Poor)	330	22.0
	3rd quantile (Middle)	300	20.0
	4th quantile (Rich)	331	22.1
	5th quantile (Richest)	257	17.1

### Knowledge of Study Participants About COVID-19

Good and poor knowledge among study respondents were found to be 841 (56.1%) and 659 (43.9%), respectively. A high number of study participants had poor knowledge about the spread of the virus through indirect hand contact and consider that no effective cure for COVID-19.

Dissimilarly, about 48.1% of the study participants were misunderstood that eating or contacting domestic animals would result in the virus. In addition, most (77.5%) of the study participants oppose knowledge items that ordinary residents should not wear general medical masks. Moreover, the vast majority of study participants were aware of the helpfulness of avoiding crowded places and isolation and treating person who contracted COVID-19 for prevention and control of the virus (Table 2).

**Table 2:** Knowledge of study participants about prevention of COVID-19, Jimma Town, Southwest Ethiopia, 2020.

**Variables**	**Response categories**	**Number**	**Percent**
COVID-19 is not seasonal flu	True	808	53.9
	Not true	692	46.1
COVID-19 spreads through droplets	True	943	62.9
	Not true	557	37.1
COVID-19 spreads through airborne	True	949	63.3
	Not true	551	36.7
COVID-19 spreads through direct hand contact	True	1,391	92.7
	Not true	109	7.3
COVID-19 spreads through indirect hand contact	True	616	41.1
	Not true	884	94.9
COVID-19 doesn't spreads through Human feces	True	38	2.5
	Not true	1,462	97.5
COVID-19 doesn't spread by insects	True	906	60.4
	Not true	594	39.6
Fever cough and SOB are hallmarks of covid-19	True	1,385	92.3
	Not true	115	7.7
Fever and cough are main clinical symptoms of covid-19	True	1,369	91.3
	Not true	131	8.7
Runny nose is less common in covid-19 patients	True	927	61.8
	Not true	573	38.2
No effective cure for covid-19	True	781	52.1
	Not true	719	47.9
Not all covid-19 pt will develop severe cases	True	1,148	76.5
	Not true	352	23.5
Eating or contacting domestic animals would result in covid-19	True	722	48.1
	Not true	778	51.9
Persons with covid-19 can infect others when fever isn't present	True	966	64.4
	Not true	534	35.6
Ordinary residents shouldn't wear general medical masks	True	337	22.5
	Not true	1,163	77.5
Children should take measures to prevent covid-19	True	1,238	82.5
	Not true	262	17.5
Individuals should avoid crowded places to prevent covid-19	True	1,056	70.4
	Not true	444	29.6
Isolation and Rx of covid-19 pt reduce spread	True	1,427	95.1
	Not true	73	4.9
observation period is greater or equal to 14 days	True	1,420	94.7
	Not true	80	5.3
Knowledge mean (±SD) score on 1–3 Likert scale		1.44 (±0.22)	
**Overall knowledgeable**	**Yes**	**841**	**56.1**
	**No**	**659**	**43.9**
Sources of information related to COVID-19	Social media	562	37.5
	TV	559	37.3
	Radio	233	15.5
	Gov't messages	91	6.1
	Family, friends	51	3.4
	News paper	4	0.3

*NB: sources of information for covid-19 can be multiple where the sum can be more than 100%. This needs checking the data!*

Analysis of data using multiple logistic regression shows that educational status and being self-employed were associated with good knowledge of study participants about COVID-19. The odds of having good knowledge among study participants; who can read and write, having primary level, had a secondary level, and having diploma and beyond educational achievements were 1.8, 2.05, 2.07, and 2.7 times higher than study respondents who had no formal education or illiterate, respectively. Whereas, the odds of having good knowledge about COVID-19 among study participants who were self-employed in their occupational status was 4.3 times higher compared to being a housewife. Also, the knowledge of study participants about COVID-19 was about 7.5 times higher among the richest study participants than the poor (Table 3).

**Table 3:** Factors affecting knowledge about prevention of COVID-19, Jimma Town, Southwest Ethiopia, 2020.

**Variables**	**Response categories**	**Knowledge**		**AOR (95% C.I.)**
		**Good, *N* (%)**	**Poor, *N* (%)**	
Sex	Male	355 (58.1)	256 (41.9)	1
	Female	486 (54.7)	403 (45.3)	0.90 (0.66, 1.23)
Age in years	<20 years	38 (55.9)	30 (44.1)	1
	20–29 years	246 (60.4)	161 (39.6)	0.89 (0.47, 1.69)
	30–39 years	288 (55.1)	235 (44.9)	0.71 (0.36, 1.41)
	40–49 years	168 (57.3)	125 (42.7)	0.77 (0.38, 1.55)
	50–59 years	74 (55.6)	59 (44.4)	0.88(0.41, 1.92)
	≥60 years	27 (35.5)	49 (64.5)	0.51 (0.21, 1.10)
Marital status	Married	664 (57.6)	488 (42.4)	1
	Single	109 (51.9)	101 (48.1)	0.75 (0.49, 1.15)
	Widowed	40 (46.5)	46 (53.5)	1.20 (0.71, 2.02)
	Divorced/ separated	28 (53.8)	24 (46.2)	1.37 (0.73, 2.57)
Educational status	Illiterate	104 (40.3)	154 (59.7)	1
	Read and write	178 (57.8)	130 (42.2)	1.79 (1.21, 2.65)^*^
	Primary school	258 (60.4)	169 (39.6)	2.05 (1.38, 3.04)^*^
	Secondary school	157 (56.5)	121 (43.5)	2.07 (1.33, 3.22)^*^
	Diploma or above	144 (62.9)	85 (37.1)	2.71 (1.59, 4.65)^*^
Occupational status	Housewife	290 (53.9)	248 (46.1)	1
	Farmer	156 (54.2)	132 (45.8)	0.85(0.55, 1.33)
	Private	68 (58.1)	49 (41.9)	1.16 (0.62, 2.14)
	Civil servant	68 (37.6)	113 (62.4)	0.59 (0.39,.89)
	Self-employee	129 (86.0)	21 (14.0)	4.26 (2.48, 7.32)^*^
	Student	98 (59.4)	67 (40.6)	1.03 (0.63, 1.69)
	Other	32 (52.5)	29 (47.5)	1.23(0.68, 2.25)
Residence	Jimma Town	693 (56.5)	534 (43.5)	1
	Suburbs	148 (54.2)	125 (45.8)	0.90 (0.61, 1.33)
Household size	≤3 individuals	192 (48.0)	208 (52.0)	1
	4–5 individuals	376 (60.6)	244 (39.4)	
	≥6 individuals	273 (56.9)	207 (43.1)	
Household RWI	1st quantile	155 (55.0)	127 (45.0)	1
	2nd quantile	164 (49.7)	166 (50.3)	0.88 (0.62, 1.24)
	3rd quantile	181 (60.3)	119 (39.7)	1.41(0.97, 2.05)
	4th quantile	117 (35.3)	214 (64.7)	0.70 (.467, 1.04)
	5th quantile	224 (87.2)	33 (12.8)	7.59 (4.57, 12.63)^*^

*Hosmer and Lemeshow Test X^2^ = 4.353, P = 0.824, df = 8*.

* *Significant association*.

### Source of Information About COVID-19

For most of the study respondents' social media (37.7%) and government-owned television (37.3%) were primary sources of information about COVID-19 followed by government-owned radio. In addition to these, government health and telecom messages were highly believed as sources of information among study respondents.

### Attitude of Study Participants About COVID-19

As shown in Table 4, most of the residents had a poor attitude (53.4%) toward the implementation of preventive measures against the COVID-19 pandemic. In this study, about 77.5 and 66.7% of the respondents had a negative attitude toward *wearing masks and care not spreading the virus* to others if they did contracted COVID-19, respectively. The majority had a good attitude towardfrequent hand washing with soap and water, use of sanitizers, and wearing a mask outside the house as a preventive measure against COVID-19. More than half (79.0%) of the study participants perceived that Ethiopia can win the battle against COVID-19. The findings indicated that 46.6% of respondents had good attitudes toward the prevention and control of the COVID-19 pandemic.

**Table 4:** Attitude of study participants about COVID-19 prevention, Jimma Town, Southwest Ethiopia, 2020.

**Variables**	**Categories**	**Number**	**Percent**
Covid-19 will finally be controlled successfully	Good attitude	1,224	81.6
	Poor attitude	276	18.4
Ethiopia can win the battle against covid-19	Good attitude	1,185	79.0
	Poor attitude	315	21.0
Takes care of contracting covid-19	Good attitude	1,052	70.1
	Poor attitude	448	29.9
Doesn't oppose wearing facemasks	Good attitude	337	22.5
	Poor attitude	1,163	77.5
Care not spread to others	Good attitude	500	33.3
	Poor attitude	1,000	66.7
Doesn't sneeze on bare hands	Good attitude	211	14.1
	Poor attitude	1,289	85.9
Covering mouth with a handkerchief when sneezing	Good attitude	1,412	94.1
	Poor attitude	88	5.9
Use the inside of the elbow when sneezing	Good attitude	1,465	97.7
	Poor attitude	35	2.3
Sanitizers can prevent you from getting covid-19	Good attitude	1,464	97.6
	Poor attitude	36	2.4
Hand wash with soap can help prevent transmission	Good attitude	1,462	97.5
	Poor attitude	38	2.5
Covid-19 can be prevented by wearing a mask outside house	Good attitude	1,454	96.9
	Poor attitude	46	3.1
Wearing a well-fitting facemask is effective	Good attitude	1,455	97.0
	Poor attitude	45	3.0
Attitude mean (±SD) score on 1–5 Likert scale		(±)	
**Over all attitude**	**Good attitude**	**699**	**46.6**
	**Poor attitude**	**801**	**53.4**

Factors like sex, age in years, marital status, educational level, residence (urban or skirt of town), and family size did not show significant association with attitude regarding COVID-19 prevention and control. With regard to variables related to good attitudes against COVID-19 we found that farmers were 1.6 times more likely to have a good attitude than housewives regarding COVID-19 prevention and control (AOR: 1.63, CI: 1.09, 2.44), however, civil servants were less likely to have good attitude compared to housewife (AOR = 0.66, CI = 0.44–0.99). An additional factor of more good attitudes against COVID-19 was study respondents in the 4th quintile of household relative wealth index had 1.5 times more likely good attitude than those in the 1st quintile (Table 5).

**Table 5:** Factors affecting attitude of study participants towards COVID-19 prevention in Jimma Town, Southwest Ethiopia, 2020.

**Variables**	**Response categories**	**Attitude**		**AOR (95% C.I.)**
		**Good, *N* (%)**	**Poor, *N* (%)**	
Sex	Male	328 (53.7)	283 (46.3)	1
	Female	371 (41.7)	518 (58.3)	0.81 (0.61, 1.08)
Age in years	<20 years	31 (45.6)	37 (54.4)	1
	20–29 years	166 (40.8)	241 (59.2)	0.99 (0.53, 1.82)
	30–39 years	237 (45.3)	286 (54.7)	1.16 (0.61, 2.23)
	40–49 years	145 (49.5)	148 (50.5)	1.20 (0.61, 2.36)
	50–59 years	75 (56.4)	58 (43.6)	1.58 (0.75, 3.31)
	≥60 years	45 (59.2)	31 (40.8)	2.02 (0.88, 4.61)
Marital status	Married	542 (47.0)	610 (53.0)	1
	Single	92 (43.8)	118 (56.2)	1.04 (0.69, 1.56)
	Widowed	43 (50.0)	43 (50.0)	1.20 (0.73, 1.97)
	Divorced/ separated	22 (42.3)	30 (57.7)	0.94 (0.52, 1.71)
Educational status	Illiterate	132 (51.2)	126 (48.8)	1
	Read and write	145 (47.1)	163 (52.9)	0.98 (0.68, 1.40)
	Primary school	213 (49.9)	214 (50.1)	1.31 (0.91, 1.89)
	Secondary school	98 (35.3)	180 (64.7)	0.81 (0.53, 1.23)
	Diploma or above	111 (48.5)	118 (51.5)	1.27 (0.77, 2.10)
Occupational status	Housewife	223 (41.4)	315 (58.6)	1
	Farmer	183 (63.5)	105 (36.5)	**1.63 (1.09, 2.44)** ^*^
	Private	57 (48.7)	60 (51.3)	1.38 (0.77, 2.47)
	Civil servant	60 (33.1)	121 (66.9)	**0.66 (0.44,.99)** ^*^
	Self-employee	64 (42.7)	86 (57.3)	0.96 (0.63, 1.46)
	Student	83 (50.3)	82 (49.7)	1.25 (0.78, 2.01)
	Other	29 (47.5)	32 (52.5)	1.03 (0.57, 1.85)
Residence	Jimma Town	530 (43.2)	697 (56.8)	1
	Suburbs	169 (61.9)	104 (38.1)	1.67 (1.19, 2.33)
Household size	≤3 individuals	172 (43.0)	228 (57.0)	1
	4–5 individuals	290 (46.8)	330 (53.2)	1.22 (0.93, 1.62)
	≥6 individuals	237 (49.4)	243 (50.6)	1.16 (0.86, 1.56)
Household RWI	1st quantile	125 (44.3)	157 (55.7)	1
	2nd quantile	133 (40.3)	197 (59.7)	0.99 (0.70, 1.40)
	3rd quantile	141 (47.0)	159 (53.0)	1.18 (0.82, 1.70)
	4th quantile	190 (57.4)	141 (42.6)	**1.50 (1.01, 2.22)** ^*^
	5th quantile	110 (42.8)	147 (57.2)	0.66 (0.43, 1.01)
Knowledgeable	Yes	400 (47.6)	441 (52.4)	1
	No	299 (45.4)	360 (54.6)	1.24 (0.98, 1.57)

*Hosmer and Lemeshow Test X^2^ = 8.244, P = 0.410, df = 8*.

* *Significant association*.

### Practice of Preventive Measures for COVID-19

In this study, most (90.5%) of the participants had no history of travel abroad or the COVID-19 hotspot area 2 months prior to the date of data collection. The mean practice score was 1.98 (± 0.319). The majority (57.5%) of the study participants had poor practice regarding COVID-19 prevention and control. Whereas, about 638 (42.5%) of the study participants had good practice toward COVID-19. During the data collection period, the study participants' practical experience of hand washing with soap and water and use of disinfectant always was 74.6 and 41.1%, respectively. The rest of them did it occasionally or never. Similarly, 52.1% of the participants had not practiced hand shaking (Table 6).

**Table 6:** Prevention practice of COVID-19 among study participants about COVID-19 in Jimma Town, Southwest Ethiopia, 2020.

**Variables**	**Categories**	**Number**	**Percent**
Travel history within 2 months	Yes	142	9.5
	No	1,358	90.5
Gone to crowded place	Always	139	9.3
	Occasionally	1,002	66.8
	Never	359	23.9
Wornmask when leaving home	Always	610	40.7
	Occasionally	840	56.0
	Never	50	3.3
Use soap to wash hands	Always	1,119	74.6
	Occasionally	374	24.9
	Never	7	0.5
When wearing a mask, test it to ensure that it fits properly	Always	904	60.3
	Occasionally	460	30.7
	Never	136	9.1
Use disinfectant to wash hands	Always	616	41.1
	Occasionally	730	48.7
	Never	154	10.3
Washhands after touching personal items of someone who has cough	Always	511	34.1
	Occasionally	482	32.1
	Never	507	33.8
Washhands after shaking hand with people who have a cough	Always	371	24.7
	Occasionally	584	38.9
	Never	545	36.3
Washhands after door handles	Always	259	17.3
	Occasionally	882	58.8
	Never	359	23.9
Refrain from getting close to those who sneeze or cough	Always	168	11.2
	Occasionally	478	31.9
	Never	854	56.9
Refrain from shaking hands of those who have cough	Always	178	11.9
	Occasionally	426	28.4
	Never	896	59.7
Refrain from often touching my nose	Always	113	7.5
	Occasionally	606	40.4
	Never	781	52.1
Practice (± SD) mean on 1–3 Likert scale		1.98 (± 0.319)	
**Overall preventive practices**	**Yes**	**638**	**42.5**
	**No**	**862**	**57.5**

As shown in Table 7, factors like sex, age, and educational status did not significantly associated with good practice regarding COVID-19 prevention and control. Regarding the good practice of COVID-19, we found that being widowed was more than half less likely to have good practice of COVID-19 than married (AOR: 0.54, CI: 0.31, 0.95) whereas good practice was two times higher among divorced or separated than married (AOR: 2.00, CI: 1.07, 3.76). Similarly, good practice of COVID-19 prevention and control was nearly half times less likely among students compared to housewives (AOR: 0.43, CI: 0.26, 0.72).

**Table 7:** Factors affecting COVID-19 prevention practices of study participants in Jimma Town, Southwest Ethiopia, 2020.

**Variables**	**Response categories**	**Practice**		**AOR (95% C.I.)**
		**Adequate, *N* (%)**	**Inadequate, *N* (%)**	
Sex	Male	246 (40.3)	365 (59.7)	0.87 (0.64, 1.18)
	Female	392 (44.1)	497 (55.9)	1
Age in years	<20 years	30 (44.1)	38 (55.9)	1.97 (0.80, 4.88)
	20–29 years	30 (44.1)	38 (55.9)	1.57 (0.81, 3.02)
	30–39 years	126 (43.0)	167 (57.0)	1.46 (0.77, 2.75)
	40–49 years	47 (35.3)	86 (64.7)	1.38 (0.72, 2.65)
	50–59 years	20 (26.3)	56 (73.7)	1.35 (0.77, 2.70)
	≥60 years	638 (42.5)	862 (57.5)	1
Marital status	Married	510 (44.3)	642 (55.7)	1
	Single	77 (36.7)	133 (63.3)	0.79 (0.52, 1.22)
	Widowed	23 (26.7)	63 (73.3)	**0.54 (0.31, 0.95)** ^*^
	Divorced/ separated	28 (53.8)	24 (46.2)	**2.00 (1.07, 3.76)** ^*^
Educational status	Illiterate	90 (34.9)	168 (65.1)	0.80 (0.47, 1.36)
	Read and write	135 (43.8)	173 (56.2)	0.82 (0.50, 1.34)
	Primary school	206 (48.2)	221 (51.8)	0.88 (0.57, 1.37)
	Secondary school	124 (44.6)	154 (55.4)	0.90 (0.57, 1.41)
	Diploma or above	83 (36.2)	146 (63.8)	1
Occupational status	Housewife	250 (46.5)	288 (53.5)	1
	Farmer	124 (43.1)	164 (56.9)	1.27 (0.82, 1.96)
	Private	48 (41.0)	69 (59.0)	0.76 (0.41, 1.43)
	Civil servant	59 (32.6)	122 (67.4)	0.74 (0.48, 1.12)
	Self-employee	87 (58.0)	63 (42.0)	1.05 (0.68, 1.62)
	Student	46 (27.9)	119 (72.1)	**0.43 (0.26, 0.72)** ^*^
	Other	24 (39.3)	37 (60.7)	1.10 (0.59, 2.07)
Residence	Jimma Town	553 (45.1)	674 (54.9)	1
	Suburbs	85 (31.1)	188 (68.9)	**0.37 (.26, 0.54)** ^*^
Household size	≤3 individuals	129 (32.3)	271 (67.8)	1
	4–5 individuals	287 (46.3)	333 (53.7)	**1.41 (1.05, 1.91)** ^*^
	≥6 individuals	222 (46.3)	258 (53.8)	**1.55 (1.13, 2.14)** ^*^
Household RWI	1st quantile	85 (30.1)	197 (69.9)	**0.31 (0.20, 0.48)** ^*^
	2nd quantile	150 (45.5)	180 (54.5)	**0.56 (0.38, 0.84)** ^*^
	3rd quantile	156 (52.0)	144 (48.0)	**0.66 (0.44, 0.98)** ^*^
	4th quantile	86 (26.0)	245 (74.0)	**0.34 (0.23, 0.50)**
	5th quantile	161 (62.6)	96 (37.4)	1
Knowledgeable	Yes	464 (55.2)	377 (44.8)	**0.36 (0.28, 0.46)** ^*^
	No	174 (26.4)	485 (73.6)	1
Attitude	Positive	288 (41.2)	411 (58.8)	1.04 (0.83, 1.31)
	Negative	350 (43.7)	451 (56.3)	1

*Hosmer and Lemeshow Test X^2^ = 8.623, p = 0.375, df = 8*.

* *Significant association*.

Moreover, study participants who were residing in the skirt of the town were 0.37 less likely to have good practice regarding COVID-19 prevention and control than those in the Town. Whereas, regarding the family size, households with a family size of four to five individuals were 1.4 times more likely to have good practice against COVID-19 compared to households with ≤3 individuals (AOR: 1.41; CI: 1.05, 1.91), similarly, the good practice regarding COVID-19 preventions and control was 1.6 times higher among the family of six or more individuals than households with ≤3 individuals (AOR: 1.55; CI: 1.13, 2.14) (Table 7).

With regards to household income, the good practice of COVID-19 prevention and control were less likely to be experienced in 1st, 2nd, 3rd, and 4th quintiles (AOR: 0.31; CI: 0.2, 0.48; AOR: 0.56; CI: 0.38, 0.84, AOR: 0.66; CI: 0.44, 0.98, AOR: 0.34; CI: 0.23, 0.50), respectively, than those study participants in 5th quintile.

### Influence of Knowledge, Attitude, and Practice

As indicated in the last columns of Table 7, those who have better knowledge of COVID-19 had less practice in its prevention and controls (**AOR: 0.36; CI: 0.28, 0.46**). Dissimilarly, attitudes toward COVID-19 did not show significant association with the practice of COVID-19 prevention and control (**AOR: 1.04; CI: 0.83, 1.31**).

## Discussion

Currently, COVID-19 is the major public health burden in the world, and the morbidity and mortality of the global community are dramatically increasing from time to time (10). Adherence to preventive measures against the virus are still the best way to reduce and control COVID-19 disease (11). The adherence to preventive measures is largely influenced by knowledge and attitude. The present study aimed at assessing knowledge, attitudes, and practice toward the prevention of COVID-19 among Jimma Town residents.

The finding of the present study indicates that only 56.1% of the study population has knowledge regarding COVID-19 disease. This much low rate of COVID-19 knowledge among Jimma residents was unexpected. Although this survey was conducted a few months after the disease was reported as a pandemic, information about the disease had been disseminated using every possible way of information transmission in Ethiopia. Because of the seriousness of the pandemic, there was overwhelming news on the pandemic everywhere.

The mean (± SD) knowledge score on the 1–3 Likert scale found in our study was 1.44 (±0.22). Yet, a high number of the study population has poor knowledge about the spread of the virus through indirect hand contact and they consider that there is no effective cure for COVID-19. Presently, several studies are conducted on KAP toward COVID-19. Compared to our findings, the majority of these studies found a higher level of knowledge regarding COVID-19. For example, a study conducted in Malaysia found an overall COVID-19 knowledge rate of 80.5% (11). This is a very high level of knowledge when compared to our findings. Another study from the same country found an overall correct knowledge rate of 91.3%, indicating a very good knowledge level (12). Likewise, a bi-national survey conducted in Egypt and Nigeria found a mean knowledge score was 14.7 ± 2.3 and about 61.6% of the population participating in the survey knew about the disease (13).

The knowledge score we found is highly lower than the knowledge score reported from Malaysian studies (11, 12). But, it is fairly lower than the bi-national survey conducted in Egypt and Nigeria (13). The reason behind the lower knowledge score of our study population may be the level of education. The authors of the bi-national survey conducted in Egypt and Nigeria argue that most of the respondents of their study had a satisfactory knowledge level of the disease and the preventive measures because both of the countries have a well-educated population (bachelor/master's degree holders). Our data also indicated that educational status is associated with good knowledge about COVID-19. The odds of having good knowledge among study participants who can read and write, had a primary level, had a secondary level, and had a diploma and beyond educational achievements were 1.8, 2.05, 2.07, and 2.7 times higher than study respondents who had no formal education or illiterate, respectively.

Social media (37.7%) and government-owned television (37.3%) were the primary sources of information about COVID-19 for the Jimma community. In addition to these, government health and telecom messages were highly believed as sources of information among them. This is similar to the report by Hager et al. and Abdelhafiz et al. (13, 14).

Regarding the attitude, the finding of our study indicated that the majority of the residents of Jimma town had a poor attitude (53.4%) toward the implementation of preventive measures against the COVID-19 pandemic. It also indicated that about 77.5 and 66.7%, respectively, of the respondents, had a negative attitude toward wearing masks and care not spreading the virus to others in case they did contracted COVID-19.

But, a study conducted in Gonder town, North Ethiopia found a better prevalence of good attitude than our finding; which reported that about 53.13% of its study population had an overall good attitude regarding COVID-19 (15). Likewise, a study conducted in Addis Ababa, Ethiopia, reported that about 60.7% of its study population had a good attitude toward the implementation of preventive measures against COVID-19 (16). These findings are fairly better than ours. Therefore, there is still an intra-country difference in Ethiopia regarding the attitude toward COVID-19. This difference could be attributed to the difference in the descriptive characteristics of the study population and the time of the study. The poor attitude about COVID-19 among our participants could also be resulted because of the low level of knowledge about the disease. Because knowledge and attitude toward COVID-19 among Jimma residents were poor, most residents were unwilling to take precautions such as wearing masks and not going to crowded places to prevent the infection by COVID-19. These potentially risky attitudes have to be removed from the Jimma community through appropriate intervention in the area.

With regard to factors affecting attitude, sex, age, marital status, educational level, residence (urban or skirt of town) and family size didn't show significant association with attitude regarding COVID-19 prevention and control. This result is contrary to a study conducted by Taddese et al. (15). In their study, Taddese et al. found the level of education and family size as the major socio-demographic factors that showed a positive association with attitude toward COVID-19. The possible reason, for the differences in the factors that affect the attitude of the communities' toward COVID-19, might be ascribed to differences in the study population, the timing of the study period, tools used to measure the variables, the sample size, the level of information exchange among the study populations.

In our study, we also found that study respondents in the 4th quintile of the household relative wealth index had a 1.5 times more likely good attitude than those in the 1st quintile. Some scholars argue that having a better income or being better in socioeconomic status enables to have resources to secure information related to COVID-19 thereby enabling to have good knowledge and a positive attitude toward the pandemic (15). This finding is an important indication for policymakers and intervention strategies to focus on community members who have no formal education.

In this study, the magnitude of good practice regarding COVID-19 was 42.5% which is a relatively small figure when compared to studies elsewhere in the world. The study participants' practical experience of hand washing with soap and water and use of disinfectant always was 74.6 and 41.1%, respectively. The rest of them did it occasionally or never. Similarly, 47.9% of the participants had been practicing hand shaking.

Compared to the majority of the studies in the literature, our study found low good practice regarding COVID-19 preventive measures. The practice of COVID-19 preventive measures in the Jimma community was lower compared to the findings from Iran (17), Nigeria (18), Lebanon (19), Venezuela (20), and Vietnam (21).

With regard to the factors affecting the outcome variables, the socio-demographic variables such as sex, age, and educational status did not significantly associate with good practice regarding COVID-19 prevention and control. But, a study conducted among a healthy Filipino population indicated that knowledge, attitude, and practice scores of their study population showed significant differences in age, sex, place of residence, occupation, and marital status (*p* < 0.000) (22). Our finding is also different from two studies conducted in Indonesia (23, 24). From this finding, we understand that the Jimma community adherence to the COVID-19 pandemic lags behind which contributed to low knowledge, attitude, and practice scores about the disease. The same finding is also reported in a study conducted in North Shoa Zone, Ethiopia (25). Hence, emphasis should be given to upraise the community's KAP regarding COVID-16 through health education and promotion.

In our population, good practice of COVID-19 was two times higher among divorced or separated than married (AOR: 2.00, CI: 1.07, 3.76). Similarly, good practice was nearly half times less likely among students compared to housewives (AOR: 0.43, CI: 0.26, 0.72), and study participants who were residing in the skirt of the town were 0.37 less likely to have good practice regarding COVID-19 prevention and control than those in the Town. Therefore, in the Jimma community, there are differences in adhering to the preventive measures of COVID-19 with respect to marital status, occupation, and residence (center of town and outskirts).

This is the first study conducted in Jimma regarding knowledge, attitude, and practice toward COVID-19. Compared to other studies conducted regarding COVID-19 in Ethiopia, the study recruited a large sample size. This can be taken as one of the strengths of the current study. Yet, it has two limitations. First, it followed a cross-sectional study design which is a single point study. Second, it was conducted at the early stage of the pandemic, which might have contributed to the low knowledge, attitude, and practice among the surveyed population about the disease. Hence, conducting follow up studies may help to find temporal changes that may be seen in the population.

## Conclusion

The findings of the current study indicate that the Jimma town community has low knowledge, attitude, and practice regarding COVID-19. But Ethiopia is a resource-challenged country. Precautionary and preventive health advisories to upraise knowledge, attitude, and practice are crucial to prevent and control the transmission of COVID-19.

In the Jimma community, knowledge, attitude, and practice scores regarding COVID-19 are significantly related to educational status, being self-employee, occupation, marital status, residence, family size, and household relative wealth index.

We hope that the current study will facilitate the implementation of effective healthcare policy by enabling healthcare officials in the area to better understand the knowledge, attitude, and practice done by the Jimma population toward COVID-19.

## Data Availability Statement

The raw data supporting the conclusions of this study will be made available by the authors, without undue reservation.

## Ethics Statement

The study protocol was approved by the Institutional Review Board of the Institute of Health, Jimma University and informed consent was obtained from each study participant.

## Author Contributions

IB, LD, AA, DY, and TG: conceptualization and data collection. IB, LD, AA, DM, DY, and TG: questionnaire preparation and validation. IB, LD, and TG: data analysis. IB, LD, AA, DM, DY, TG, and YW: original draft preparation. LD, AA, DM, and DY: resource and supervision. All authors have read and approved the final manuscript before submission.

## Funding

The study was funded by Jimma University (Funding Number: IHRPGD228/2020). The funding body was not involved in the design of the study, data collection, analysis, interpretation of data, and writing the manuscript.

## Conflict of Interest

The authors declare that the research was conducted in the absence of any commercial or financial relationships that could be construed as a potential conflict of interest.

## Publisher's Note

All claims expressed in this article are solely those of the authors and do not necessarily represent those of their affiliated organizations, or those of the publisher, the editors and the reviewers. Any product that may be evaluated in this article, or claim that may be made by its manufacturer, is not guaranteed or endorsed by the publisher.

## Footnotes

^1^ https://www.who.int/publications/m/item/weekly-operational-update-on-covid-19-30-november-2021
